# Fatty Acids in Cnidaria: Distribution and Specific Functions

**DOI:** 10.3390/md23010037

**Published:** 2025-01-13

**Authors:** Vasily I. Svetashev

**Affiliations:** A.V. Zhirmunsky National Scientific Center of Marine Biology, Far Eastern Branch, Russian Academy of Sciences, ul. Palchevskogo 17, Vladivostok 690041, Russia; vsvetashev@mail.ru

**Keywords:** Cnidaria, Octocorallia, Hexacorallia, corals, fatty acids, uncommon fatty acids, PUFA, symbiosis, zooxanthellae, food chain markers

## Abstract

The phylum Cnidaria comprises five main classes—Hydrozoa, Scyphozoa, Hexacorallia, Octocorallia and Cubozoa—that include such widely distributed and well-known animals as hard and soft corals, sea anemones, sea pens, gorgonians, hydroids, and jellyfish. Cnidarians play a very important role in marine ecosystems. The composition of their fatty acids (FAs) depends on food (plankton and particulate organic matter), symbiotic photosynthetic dinoflagellates and bacteria, and de novo biosynthesis in host tissues. In cnidarian lipids, besides the common FA characteristics of marine organisms, numerous new and rare FAs are also found. All Octocorallia species and some Scyphozoa jellyfish contain polyunsaturated FAs (PUFAs) with 24 and 26 carbon atoms. The coral families can be distinguished by specific FA profiles: the presence of uncommon FAs or high/low levels of common fatty acids. Many of the families have characteristic FAs: Acroporidae are characterized by 18:3n6, eicosapentaenoic acid (EPA) 20:5n3, 22:4n6, and 22:5n3; Pocilloporidae by 20:3n6, 20:4n3, and docosahexaenoic acid 22:6n3 (DHA); and Poritidae by arachidonic acid (AA) and DHA. The species of Faviidae show elevated concentrations of 18:3n6 and 22:5n3 acids. Dendrophylliidae, being azooxanthellate corals, have such dominant acids as EPA and 22:5n3 and a low content of DHA, which is the major PUFA in hermatypic corals. The major and characteristic PUFAs for Milleporidae (class Hydrozoa) are DHA and 22:5n6, though in scleractinian corals, the latter acid is found only in trace amounts.

## 1. Introduction

The phylum Cnidaria is an ancient member of Metazoa, with a simple body organization. It comprises a diverse group of relatively primitive diploblastic animals characterized by having an extremely complex cellular organelle referred to as cnida (which in ancient Greek means “stinging nettle”) or nematocysts. Cnidarians have a characteristic radial symmetry and the planula and polyp stages in their ontogeny [1].

The phylum Cnidaria consists of nearly 12,500 extant species from the classes Anthozoa, Medusazoa, Hydrozoa, and Scyphozoa [2]. Cnidarians play a very important role in marine ecosystems, of which tropical coral reefs are exceptionally productive [3]. The high levels of production in reef-building corals are achieved primarily due to their symbiotic algae. Zooxanthellae (unicellular algae) in corals account for 50–70% of the total primary production in most reefs. The number of coral species at depths greater than 50 m is usually higher than that on shallow, tropical coral reefs [4]. Jellyfish are attracting increasingly more attention because of the frequently documented cases of their mass proliferation, their potential capability of shaping the food web structure [5], and their importance in global biogeochemical cycles [6]. Cnidarian jellyfish are considered a sustainable source of high-value compounds that can find biotechnological applications, e.g., as antioxidants and nutraceuticals [7,8].

The lipid biochemistry of Cnidarians deserves special attention. The use of thin-layer chromatography (TLC) has shown that their total lipids contain phospholipids, sterols, free fatty acids (FAs), triacylglycerols (TGs), monoalkyl-diacylglycerols (MADAGs), and wax esters (WEs). The content of free FAs in total lipids is usually low, approximately 1.0–1.5%. The concentrations of storage neutral lipids such as TG, WE, and, less commonly, MADAGs vary between 20 and 30, 30 and 50, and 5 and 10%, respectively. The concentrations of structural lipids, sterols, and phospholipids (PLs) range between 5 and 10, and 10 and 20%, respectively [9]. Lipids of the sea anemone *Anthopleura elegantissima* have been found to include an unusual phosphosphingolipid with a 2-aminoethylphosphonic group, ceramide aminoethylphosphonate (CAEP) [10]. The latter, in turn, has been found as a major phospholipid (up to 20%) in gorgonarians and alcyonarians [11,12]. In the cold-water alcyonarian *Gersemia rubiformis*, the content of CAEP and N-methyl-CAEP reaches 29% of total phospholipids [13]. A possible role of CAEP is the stabilization of its own membranes against toxic actinoporins, which constitute the main family of pore-forming proteins from sea anemones [14]. In jellyfish, ceramide 2-aminoethylphosphonate is concentrated in the membranes of the tentacles (oral arms) bearing stinging cells, where it may resist hydrolysis due to the endogenous phospholipase A2 [15]. In this review, we compiled data on the FAs of the major Cnidaria taxa, with a special focus on characteristic components, marker FAs, the presence of symbionts, and new and uncommon fatty acids. It is relevant to note here that the commonly applied term ‘coral’ has no valid taxonomic definition. “Initially, the Mediterranean coral, *Corallium rubrum* (Anthozoa: Octocorallia), having a calcitic skeleton was referred to as ‘coral’. Afterwards, the term was used not only for other octocorallians, such as the blue coral (Heliopora), but also for hexacorallians, such as black corals with horny skeleton, *Antipathes*, or reef-building corals (scleractinians or Madreporaria) with aragonitic skeleton, or even hydrozoans such as fire corals (Millepora). However, ‘coral’ is also used to designate Octocorallia without hard skeleton, alcyonarians (soft corals), which adds even more confusion. Scleractinian corals are known for their skeleton and are, in this case, called ‘reef-building corals’ or hermatypic corals (‘herm’ in ancient Greek means ‘reef’). Hermatypic corals host unicellular symbiotic dinoflagellates (*Symbiodiniactaea*), commonly referred to as zooxanthellae, in their tissues. However, all scleractinians are not reef builders (ahermatypic corals), although they are characterized by their aragonitic skeleton” [16].

In the literature, different abbreviations are commonly used for the structures of FAs, e.g., 18:1ω9, 18:1n9, and 18:1n-9 for oleic acid, which are equivalent. Eicosapentaenoic acid (EPA) 20:5(n-3) in texts can be abbreviated as 20:5(n-3), 20:5n3, and 20:5ω3. Sometimes, abbreviations are used to indicate the position of the double bond counted from the carboxyl end, e.g., Δ7-18:1, Δ9,12-18:2, and Δ5,8,11,14,17-20:5. In this review, I will use the most simple abbreviations with one letter, like 20:5n3.

## 2. Some Methodological Notes

Comparing various data on FA composition that are scattered in numerous articles and obtained by different analytical protocols poses a major challenge. In some cases, it may lead to erroneous or not fully reliable results. The most common mistakes in marine FA analyses are the too short time of the GC run and the use of saponification or the basic transesterification of microalgae lipids.

### Total Lipid Extraction and FA Analysis

**Figure 1:**
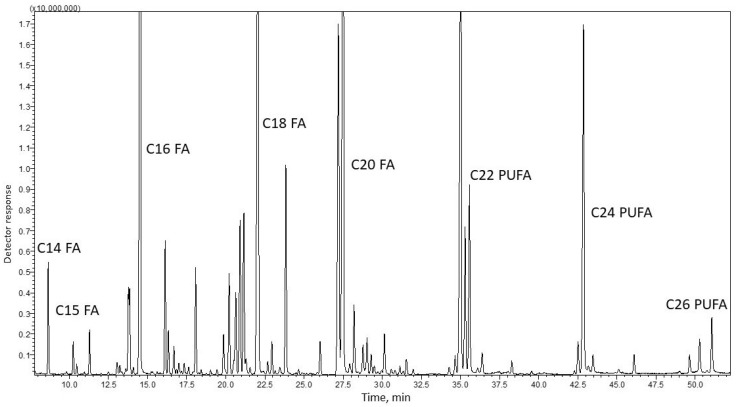
GC-MS chromatogram of medusa *Rhopilema esculentum* total lipid FAMEs on SLB-5ms column.

Since some Cnidaria species (especially jellyfish) contain only ca. 0.2% total lipids (TLs), it is better to use the cost-effective method proposed by [17] for lipid extraction. For the isolation of TLs from stony corals, the extraction of crashed corals, instead of the preliminary isolation of soft tissues by air spraying, is a much more efficient approach [18]. For the preparation of FA methyl esters (FAMEs), the use of acid reagents (5% HCl or 1–2% sulfuric acid in methanol), 50 °C overnight or 80 °C for 90 min, is more preferable [19] because the preliminary saponification or transesterification with NaOCH_3_ in methanol induces the decomposition and isomerization of octadecapentaenoic acid 18:5n3 into four isomers [20,21]. The methyl ester of octadecapentaenoic acid 18:5n3 on GC, separated on a Supelcowax 10 polar column, has an equivalent chain length (ECL) of 20.22, while isomers run much more latter, with ECL of 20.48, 20.88 (main peak), and 21.19. Accordingly, instead of the peak of 18:5n3 with an ECL of 20.22, which is located close to (even overlaps) the peak of 20:1n9, one can see artifact peaks with an ECL of 20.88. Identifying 18:5n3 on a non-polar column with 5% phenyl silicon such as SLB-5 or one similar, where the ECL is equal to 17.55, is much easier. For the identification of marine-derived FAs, FAME standard mixtures such as Supelco 37 are often used, but these do not contain the usual marine FAMEs such as 18:1n7, 18:4n3, 20:4n3, 22:5n3, and 22:5n6. Moreover, marine organisms may have PUFAs with C24 and C26 and even longer carbon chains. There are reports about the presence of elaidic acid (18:1n9 trans), which is not characteristic of marine samples. Besides the use of sophisticated equipment like GC–MS, there is a simple and convenient method of FAME identification carried out by the calculation of ECL values. It helps identify the most common and unusual FAs merely by comparing its own data with published tables of ECL values, e.g., in [22]. Moreover, it allows for the calculation or prediction of ECL for new FAs [23]. More details on ECL values for FAMEs on columns with different phases and their MS spectra are available at Chrombox.org [24]. For very-long-chain PUFAs with the carbon chains C24–C26, which are characteristic of the class Octocorallia, the analysis is preferably conducted under special conditions because these FAs have very long retention times (RTs). For example, on the common polar column Supelcowax 10 at 220 °C, the RT for the acids 22:6n3, 24:6n3, and 26:7n3 are 28.4, 48.1, and 86.7 min, respectively. On the non-polar column SLB-5ms with a temperature ramp of 170–280 °C (2°/min), the RT is quite moderate, <55 min, and the separation is good. An example of a chromatogram for total lipids in the jellyfish *Rhopilema esculentum* is shown in Figure 1.

## 3. Fatty Acids of Hexacorallia

The class Hexacorallia comprises scleractinian, black corals, tube anemones, and sea anemones (with a total of nearly 4300 species) [1]. The major orders are Actiniaria (ca. 1200 species), Antipatharia, Ceriantharia, Corallimorpharia, Scleractinia (ca. 1300 species), and Zoantharia.

### 3.1. Fatty Acids of Actiniaria

Actiniaria are soft-bodied, solitary polyps with tentacles. They are predatory animals. In Actiniaria species, the major PUFAs are DHA, 22:5n3 (DPA3), EPA and 22:4n6, while n3 FAs prevail over n6 acids (with an n6/n3 ratio of 0.1–0.80) (Table 1). The content of arachidonic acid is 2.6–7.7% and is always lower than that of EPA. The total amount of C22 PUFA is greater than that of C20 PUFA. Abyssal and cold-water species accumulate monoenoic C20:1 and C22:1 acids. Together with marked concentrations of 18:1n9 and DHA, these indicate feeding on zooplankton. Of particular interest is the high concentrations of n3 docosapentaenoic acid and 22:4n6, which are homologs of arachidonic and eicosapentaenoic acids.

In [32], the authors compared the effects of symbiotic dinoflagellates (*Symbiodinium muscatinei*) and chlorophytes (*Elliptochloris marina*) on lipids of the common sea anemone, *Anthopleura elegantissima*. In general, the authors noted an increased total concentration of FAs in *A. elegantissima* with symbionts, but the results were not complete (because a standard MEFA Supelco 37 mixture lacks some common marine acids such as 18:4n3, 18:5n3, and 18:1n7). In [33,34], a set of new FAs, including non-methylene interrupted (NMI) dienoic and trienoic acids C18–C23, brominated FA, etc., were identified in phospholipids. In abyssal Actiniaria species, the uncommon FA 21:4n7 was detected at 2.3% and tetracosahexaenoic acid (TPA) 24:6n3 at 0.8% [35]. In a discussion of the first communication on tetracosapolyenoic acids in Cnidaria [36], the authors mentioned that “among all other coelenterates studied (class Hydrozoa: order Leptolida: class Hexacorallia: orders Actiniaria, Zoantharia, Ceriantharia, insignificant amounts of TPA were found (up to 1%)”. In [37], the FA compositions of three species of bathyal sea anemones were studied, and high proportions of mono- and polyunsaturated fatty acids (MUFAs and PUFAs), as well as n3 FAs, and unusually low proportions of arachidonic acid were found. High values of 20:5n3, 20:1n9, and 22:1n11 suggested feeding on zooplankton. In [38], low contents of DHA in neutral lipids (NLs) and PLs (0.4–2.6%) and AA (2.7–4.7%) were reported for the red and green forms of the Black Sea *Actinia equina*. In the paper, the structure and marked concentrations of the less common fatty acids 22:4n6 and 22:5n3, previously found in another sea anemone species, were well described.

### 3.2. Fatty Acids of Antipatharia and Zoantharia

The major PUFAs in black thorny coral (*Stauropathes arctica*), which is a cold-water antipatharian, are fatty acids of the n3 family, 22:5n3 and EPA, constituting 17.1 and 10.6%, respectively [39], (Table 2). Arachidonic acid (AA) and DHA, which are common for Cnidaria, have only minor proportions, 1.5 and 1.6%, with a low n6/n3 ratio (0.11). The proportion of saturated acids is also low, 10%, while that of the monoenoic acids C20:1 and 22:1 is 34.5%. This suggests a mainly zooplankton-based diet. In *Palythoa* [40], octadecapentaenoic acid has been detected, which, together with 18:3n6 and 18:4n3, clearly indicates the presence of zooxanthellae.

In zoantharian species of the genus *Palythoa*, the major PUFAs are AA, DPA3, 22:4n6, and AA. The contents of EPA and DHA are low, as well as those of C20:1 and 22:1 acids. In [42], a statistical analysis of the distribution of 10 major PUFAs was carried out for 66 hexacoral and octocoral species. For nine species of *Palythoa* and *Zoanthus*, the Zoanthidae, the data were similar to those presented in Table 3. In [40,41], the uncommon acid 18:5n3, characteristic of symbiotic dinoflagellates, was found. Thus, the detection of the acids 18:3n6, 18:4n3, and 18:5n3 indicated the presence of symbiotic dinoflagellates [43]. In [41], two azooxanthellate species of *Palythoa* showed AA as a major fatty acid (8–10%), much lower levels of EPA and DHA (ca. 2%), and noticeable amounts of 22:4n6 (ca. 3.5%) and 22:5n3 (ca. 5%). The authors of [42] considered concentrations of only five major n3 and five n6 acids.

### 3.3. Order Scleractinia

According to [44], this order comprises 1363 extant species from the major families Acroporidae (270 species), Agariciidae (47), Caryophylliidae (307), Dendrophylliidae (187), Fungiidae (54), Lobophylliidae (60), Merulinidae (152), Pocilloporidae (55), Poritidae (100), and Turbinoliidae (70). Initially, the data on the FA composition of scleractinian corals were somewhat contradictory and incomplete. The first screening of FAs in Caribbean Scleractinia was conducted by [45]. However, since there were some drawbacks in the lipid isolation method and FAME analysis, the data presented have only a limited value. Later on, the authors of the study [46] compared the PUFA contents and compositions between eight species of corals from the depths of 2–5 and 25–30 m and suggested that the relatively high levels of PUFAs, such as 22:5n3 and 22:6n3 in the species from the depths of 25–30 m, compared to those in shallow-water species, might be derived from food (copepods), while for the species from the depths of 2–5 m, the main source of FAs might be symbiotic zooxanthellae. In [47], a more advanced FA analysis method was applied for Scleractinian corals from the families Acroporidae, Pocilloporidae, Poritidae, and Dendrophylliidae from Vietnam and the Seychelles (Table 3).

**Table 3:** FA composition (% of total FAMEs) of scleractinian corals from the families Acroporidae, Seriatoporidae, Poritidae, and azooxanthellate Dendrophylliidae from Vietnam (V) and the Seychelles (S) [47].

Fatty Acids	*Acropora* *nasuta*	*Acropora* *millepora*	*Acropora* *florida*	*Seriatopora* *caliendrum*	*Stylophora* *pistillata*	*Stylophora* *pistillata*	*Pocillopora* *damicornis*	*Pocillopora* *verrucosa*	*Porites* *lutea*	*Goniopora* sp.	*Tubastrea* *coccinea ^a^*
	V	V	V	S	S	V	V	V	V	V	S
16:0	38.6	24.5	33.1	24.0	21.8	41.0	44.5	41.7	49.1	17.2	7.2
16:1n7	1.0	1.0	1.2	2.1	2.9	3.0	3.1	2.3	1.9	3.5	5.9
18:0	7.3	9.3	9.0	6.5	4.6	10.6	10.2	8.0	7.2	5.7	4.2
18:1n9	8.0	2.2	3.2	13.3	14.4	5.5	4.9	7.0	3.8	11.7	23.3
18:3n6	5.8	9.5	8.2	2.7	3.1	5.4	4.1	2.6	9.7	4.5	0.4
18:4n3	2.6	6.6	5.1	1.7	1.3	1.4	0.8	3.3	2.9	2.3	0.7
20:1	0.7	1.1	1.0	0.9	0.7	1.4	1.1	2.6	0.9	5.9	3.0
20:3n6	1.9	2.4	0.3	11.3	12.3	7.2	7.6	3.2	1.6	3.6	0.8
20:4n6	7.1	7.2	11.0	4.8	4.3	1.7	2.0	1.8	2.3	13.3	7.8
20:5n3	0.8	10.4	6.9	2.6	2.0	1.4	1.4	3.2	3.3	4.1	14.9
22:4n6	4.3	6.0	6.3	1.5	1.8	1.0	0.9	1.3	1.4	3.3	4.7
22:5n3	0.9	3.0	1.2	1.2	1.3	4.5	0.4	0.7	0.8	1.0	16.4
22:6n3	10.8	12.6	6.7	16.9	16.4	8.8	9.5	10.4	5.3	15.7	1.4
PUFA	40.4	59.6	49.4	48.5	50.4	31.5	30.5	30.0	29.3	53.8	52.0
(n3/n6)	0.6	1.2	0.7	0.9	1.0	0.7	1.2	1.7	0.8	0.8	2.1

*^a^* Azooxanthellate species.

There the major FAs were 16:0, 18:0, 18:1n9, 20:4n6, 20:5n3, 22:4n6, and 22:6n3. Some of the coral families had significant levels of characteristic FAs: 20:3n6 for Pocilloporidae, 18:3n6, 18:4n3, and 22:4n6 for Acroporidae, and 18:3n6 for Poritidae. The noticeable amounts of 18:3n6 and 18:4n3 indicated the input of symbiotic microalgae. Two asymbiotic species of *Tubastraea*, from the family Dendrophylliidae, showed the major FAs 18:1n-9 (23.3–26.4%), EPA, AA, and 22:5n3. The concentration of DHA, 18:4n3, and 18:3n6 in these species was low, nearly 2%. Of particular note was that the azooxanthellate species of *Tubastraea* had high concentrations of AA and EPA and their C22 homologs, 22:4n6 and 22:5n3. The contents of some common acids such as 18:1n7, 18:2n6, 20:1n7, 20:2n6, and 22:2n6 were no higher than 2%. The content of branched- and odd-chain acids of bacterial origin was also insignificant. In addition, the authors found differences in the FA composition between the same species from different geographic places and depths of sampling. An even more extensive FA study of 16 species of reef-building corals was published later [48]. There, in addition to the above-mentioned families, corals of Faviidae, Pectiniidae, and Fungiidae were also analyzed (Table 4).

In general, the data reported by [9,47,48] were similar. In two *Favia* species, the major PUFAs 18:3n6, AA and 22:5n3 and a low level of EPA were found. In *Balanophillia* sp., in the family Dendrophylliidae, 18:1n9 was a major acid (20.3%). The polyunsaturated FAs were DHA (9.2%), AA (3.4%), DPA3 (2.3%), and 18:3n6 (2.4%). The levels of the rest of C18–C22 PUFAs were no higher than 2% each.

**Figure 2:**
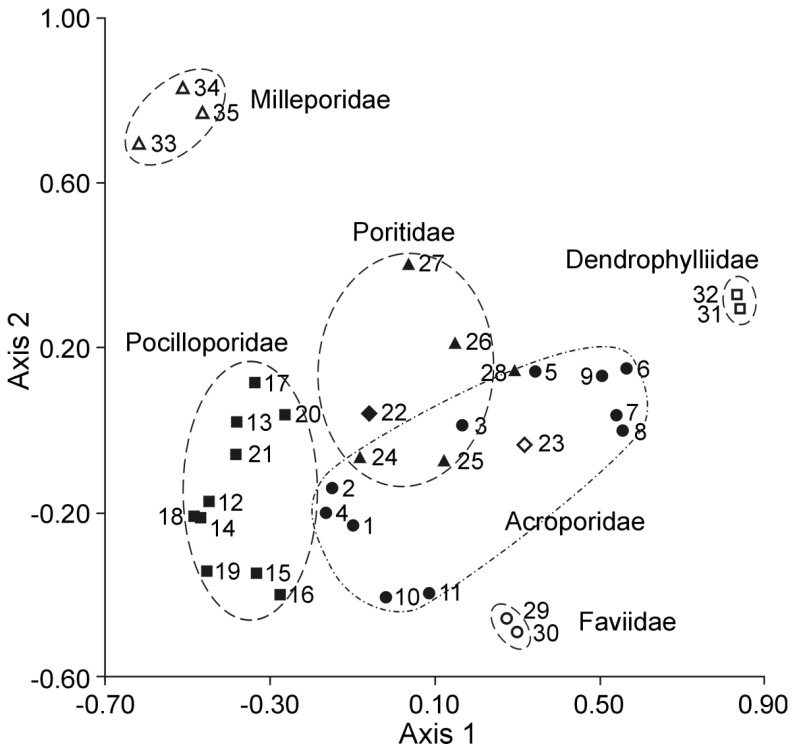
Multidimensional scale analysis performed using ten FAs selected as marker variables. Filled circles are for Acroporidae; filled squares for Pocilloporidae; filled triangles for Poritidae; open circles for Faviidae; open squares for Dendrophylliidae; filled rhombi for Pectiniidae; and open rhombi for Fungiidae. The plot is redrawn from [48].

The most noteworthy results were obtained through the statistical processing of FA analysis data. In [47,48], principal component analysis (PCA) was performed, using 10 variables (the square root of the selected unsaturated fatty acid content), of the FAs 18:1n-9, 18:1n-7, 20:1n9, 20:1n7, 20:4n3, 20:5n3, 22:4n6, 22:5n3, and 22:6n3. Figure 2 shows the relationships between the FA compositions and taxonomy of 35 reef-building coral specimens. The four families of corals can be recognized in the score plot.

## 4. Octocorallia

### 4.1. Alcyonacea

The FA composition of the families Alcyoniidae and Nephtheidae was analyzed in [48] and are here presented in Table 5. The polyunsaturated FAs 24:5n6 and 24:6n3 (TPA) were the most characteristic of all Alcyonacea, comprising 3.1–10.03% in them. The highest level of TPA was found in the azooxanthellate species of *Dendronephthya*. The major FAs were 16:0 (up to 35%), arachidonic acid (9.4–19.7%), and 18:3n6 (up to 10.6%). In all species, the EPA content was only 2.1–5.9%, and the DHA content was 2.1–6.9%. The n6/n3 ratio was always >1 (Table 6). Most species had high concentrations of the uncommon fatty acid 16:2n7 (0.3–9.4%). The contents of the C20:1 and C22:1 fatty acids, which indicate feeding on zooplankton, were low. In most species, there were marked concentrations of the C18 PUFAs 18:3n6 and 18:4n3, which are typical of symbiotic zooxanthellae [43]. Two (zooxanthellate and azooxanthellate) species of Nephtheidae had a difference in the contents of 18:3n6 (7.9–0.40%), EPA (5.9–1.4%), and TPA (6.4–10.3%). It was reported that the main sources of the fatty acids 16:2n-7, 20:4n6, and 20:5n3 were, respectively, protists, zooplankton, and phytoplankton that corals fed on [49]. Obviously, zooxanthellae was the main food source for Alcyonacea. Only *Lobophytum* and *Sarcophytum* had noticeable levels of bacterial FAs (8.3 and 3.9%). These data generally supported the results of previous studies where 10 *Dendronephthya* species had been analyzed (the major FAs were AA (15.3%) and TPA (17%)) [48].

### 4.2. Gorgonacea

For Gorgonacea species, the absolute dominance of arachidonic acid (up to 47% in *Echinogorgia* sp.) and high levels of C24 PUFA were typical (Table 6). The azooxanthellate species *Leptogorgia piccola* had the highest content of TPA, 21.1%. Other major FAs were 16:1n7, 20:5n3, 22:5n6, 22:6n3, 24:5n6, and 24:6n3. The content of common C18 and C20 acids such as 18:2n6, 18:4n3, 20:1n9, and 20:3n6 was no higher than 2%. Bacterial odd- and branched-chain FAs were detected in quite noticeable amounts (up to 5.2% of total FAs). The average ratio of oleic to *cis*-vaccenic acids, 18:1n9/18:1n7, was 17.9 and 1.5 for zooxanthellate and azooxanthellate species, respectively. Thus, the zooxanthellate species of *Rumphella* showed marked levels of 18:3n6 and 18:4n3, characteristic of endophotosymbionts. The FAs of the n6 series were dominant PUFAs in the species under study; the n6/n3 ratio ranged from 2.7 to 17.1 with an average of 7.0. I did not find any significant difference between zooxanthellate and azooxanthellate coral species in the n6/n3 ratio. Large amounts (up to 10%) of uncommon furanoic FAs (F-acids, containing a furan ring in the hydrocarbon chain) were detected in azooxanthellate Gorgonacea [51].

**Table 6:** Main FA composition (% of total FAs) of zooxanthellate and azooxanthellate Gorgonacea species from the families Melithaeidae, Acanthogorgiidae, Nidaliidae, Plexauridae, Elliselliidae, Paramuriseidae, and Gorgoniidae [51] and data for *Leptogorgia piccolo* [52].

Fatty Acids	*Acabaria* *erythraea*	*Acanthogorgia* *isoxia*	*Chironephthya* *variabilis*	*Echinogorgia* sp.	*Ellisella* *plexauroides*	*Menella* *praelonga*	*Leptogorgia* *piccola*	*Rumphella* *aggregate* ^Z^
16:1n7	1.10	2.10	1.50	1.60	1.07	1.87	4.8	2.65
16:0	7.50	11.87	11.07	14.00	8.90	8.83	9.7	32.95
18:3n6		0.10				0.10	0.6	1.10
18:4n3	0.40	0.17	0.10		0.13	0.27		2.15
18:2n6	0.90	1.03	1.37	0.70	0.90	0.83	1.0	0.65
18:1n9	2.40	3.27	4.03	3.10	2.67	2.13	2.5	3.85
18:1n7	1.90	2.13	1.63	2.20	2.53	1.93	3.2	0.35
18:0	6.50	6.17	5.80	5.30	7.40	5.30	7.0	9.60
20:4n6	37.20	38.77	40.43	47.60	39.30	39.70	20.5	13.15
20:5n3	1.70	3.27	1.90	2.20	1.97	3.67	8.0	2.05
22:5n6	5.70	1.13	0.70	0.20	0.90	1.27	1.5	0.10
22:6n3	3.90	2.53	1.37	0.80	2.90	2.60	3.8	1.60
22:4n6	0.60	3.83	0.70	0.40	8.97	3.93	2.8	0.65
24:5n6	14.50	7.50	12.33	8.90	3.10	9.13	15.8	3.40
24:6n3	2.30	2.40	1.30	2.60	1.40	2.93	5.3	0.20
Sum TPA	16.8	9.90	13.63	11.50	4.50	12.06	21.1	3.60
Odd+Br	2.50	4.53	5.67	5.20	5.17	4.20	2.3	3.0
20:1+22:1	0.60	1.60	1.05		1.30	1.85		0.60

^Z^ Zooxanthellate species.

Table 7 shows the FAs of azooxanthellate, the zooxanthellate species of Gorgonacea, and gorgonarian *Bebryce* sp. with a sponge symbiont [51]. The arachidonic (20:4n6) and palmitic (16:0) acids were dominant in all the species. However, the azooxanthellate species had a 2–3-fold lower content of 16:0. The arachidonic acid content was 40.5%, and the sum of n6 acids reached 54.5% of total FAs in the azooxanthellate species. The contents of EPA and DHA were nearly 2.5%. Bacterial odd- and branched-chain FAs were detected in quite a noticeable amount, up to 6.1% of total FAs, in *Bebryce* sp. The total content of tetracosapolyenoic acids was substantial in all species and reached 11.4% in the azooxanthellate species. *Bebryce* sp. with a symbiotic sponge had 17.2% of demospongic acids with C25, C26, and C28 carbon atoms, which are major FAs in marine and fresh-water sponges of the class Demospongiae [53].

### 4.3. Helioporacea and Stolonifera

Helioporacea comprises only two monogeneric families that are unique among octocorals in producing calcified skeletons of crystalline aragonite. The well-known blue coral, *Heliopora coerulea*, is distributed widely across the Indo-Pacific where it is a common member of shallow coral reef communities. The enigmatic genus *Epiphaxum* is known from only a few localities at depths of 50–400 m. The phylogenetic relationships of these two families to one another and to other Octocorallia remain uncertain [1]. The FAs in *H. coerulea* have been analyzed only in two studies [48,54] (Table 8), whose data look contradictory: one sample has 18:3n6 as the major PUFA. Furthermore, both samples demonstrated the lowest level (0.2–0.9%) of arachidonic acid found in Octocorallia. Another feature of the FAs from *H. coerulea* is the lowest content of tetracosapolyenoic acids, with approximately 2% of 24:6n3.

The major PUFAs in *Carijoa riisei* (azooxanthellate) and *Clavularia* sp. are AA, 18:4n3, EPA, DHA, and 24:5n6. Of particular interest is the high concentrations of FAs 18:3n6 and 18:4n3, which, together with 18:5n3, are typical of symbiotic zooxanthellae. The study [42] showed AA as the major PUFA (24.2%) and EPA, 22:4n6, and DHA, with levels of nearly 3% each in *C. riisei*. In the cited article, data on only the five major n3 and five n6 PUFAs were presented.

### 4.4. Pennatulacea

Sea pens, or pennatulaceans, are a highly specialized group of Cnidaria. They are benthic sessile animals adapted to living partially buried in sediment. Sea pens are encountered all over the world’s oceans and seas and at almost all depths (from the intertidal zone to depths greater than 6100 m). Many deep-sea species have nearly cosmopolitan distributions in such habitats [55]. Sea pens are colonial animals with multiple polyps possessing eight tentacles. A single polyp develops into a rigid, erect stalk (rachis) and loses its tentacles, forming a bulbous ‘root’ or peduncle at its base.

There are plenty of data on the FA composition of Pennatulacea species, but most publications do not show the presence of the tetracosapolyenoic acids 24:5n6 and 24:6n3, which are major components (constituting up to 20% in total) (Table 9). In deep-sea species, the prominent components were EPA, C20:1 and 22:1 acids, with n3 > n6 in most samples, but in the shallow-water *Rennilla koellikeri*, a high content of AA was found. In all species, the DHA content was low (within the range of 0.5–3.4%), and only in the NLs of the deep-sea *Pavonaria finmarchica* did it reach 9.4%. Information on the FAs of Pennatularia species is generally insufficient and somewhat contradictory. One of the issues is the use of different analytical protocols. In [41], the FAs of pennatulaceans had higher contents of 20:5n3, 22:5n3, 22:4n6, and 24:6n3 and lower contents of 18:4n3 and 24:5n6 compared to those of alcyonaceans. Also, the authors found a similarity of the FA profile with that of the azooxanthellate *Pseudopterogorgia* sp. [39] and carried out an extensive comparative analysis of FAs (without TPAs, as they used a too short analysis time) in cold-water Cnidaria, including 25 samples of sea pens, 16 samples of Gorgonians, and 32 samples of soft corals. Soft corals and gorgonians (the order Alcyonacea) were close in composition and likely fed on phytodetritus resulting from algae, macrophytes and/or foraminifera, while sea pens (the order Pennatulacea) seemed to consume more diatoms and/or zooplankton. The study [56] described the occurrence of TPAs in the stenophagous nudibranch mollusk *Armina maculata* that feeds exclusively on the sea pen *Veretillum cynomorium*. There is also experimental evidence that TPAs are retained in tissues of *A. maculata,* even after 30 days of starvation [57]. In abyssal Pennatulacea species, the uncommon acid 21:4(n-7) was found [35].

## 5. Medusozoa

### 5.1. Cubozoa, Order Cubomedusae

Cubozoa is a class of the phylum Cnidaria currently comprised of only 36 valid species [1]. Cubozoans are also known as box jellyfish, usually small in size. Their tentacles are located at the corners of the square umbrella margin. The venom of cubozoans is lethal to humans. The data on cubozoans’ lipids and FAs are limited to a relatively old communication [60]. Four analyzed species showed similar FA compositions. The major PUFA were n3 family: 22:5n3, EPA, and DHA (Table 10). The content of AA was low; the level of n6 acid was increased due to the presence of the less common FA 22:4n6 only in *Maeotias inexpectata*. According to the published database of FA microalgae [61], significant amounts of the fatty acid were found in microalgae from Prymnesiophyceae, Cryptophycea, and Bacillariophyceae. These microalgae are likely used as food or/and symbionts. The contents of C18 PUFA and C20:1 and C22:1 acids were generally low, 2–5 and 1.1–2.0%, respectively.

### 5.2. Hydrozoa

Hydrozoa is a speciose class of Cnidaria comprised of more than 3500 species. They form colonies of polyps and free-swimming medusa [1]. Most hydrozoans are predators or filter feeders. A few species have symbiotic zooxanthellae. Colonial polyps can secret chitinous or calcareous coatings similar to that in Scleractinia.

#### Family Milleporidae

Corals of the genus *Millepora* are known as fire coral for their painful stings to humans. They look like Scleractinia corals but are related to Hydrozoa. The species are characterized by the dominance of C22 PUFA 22:4n6, 22:5n6, and 22:6n3 and low levels of AA and EPA (Table 11). The n3/n6 ratio varies between 2.0 and 5.1. The fatty acids of *Millepora* species and scleractinian reef-building corals differ in many features (Table 4). The samples from Okinawa [62] had unexpectedly very low concentrations of PUFA, only 21.4%. This can be explained by the accumulation of neutral lipids with a high concentration of saturated and monoenoic acids.

### 5.3. Families Physaliidae and Velellidae

The Portuguese man-of-war *Physalia physalis* (the family Physaliidae) and the wind sailor *Velella velella* (the family Velellidae) are two pleustonic hydrozoan species. *Physalia physalis* inhabits tropical and subtropical waters, while *V*. *velella* occurs in warm and temperate waters. In both species, DHA, EPA and 18:4n3 as major PUFAs and a very low content of n6 acids were found, while the n3/n6 ratios were 10.8 and 12.4 [63] (Table 12). The presence of C18 PUFA suggested the existence of zooxanthellae. The difference between the two species was not significant. However, previous data on *Physalia* FAs [64] and unpublished results showed arachidonic acid as a major component, and the n3/n6 ratios were only 1.0 and 1.7. In all species, the contents of C20:1 and C22:1 and odd- and branched-chain acids were low.

### 5.4. Hydromedusae

Hydromedusae are a diverse group (>800 species worldwide), in which most representatives are <10 mm in size. All species are carnivorous, capturing prey with specialized stinging cells referred to as nematocysts. Most hydrozoans show an alternation between the polyp and medusa phases. The difference between most hydrozoans and scyphozoans is that the polyp stage in the former usually predominates, with medusae small in size or sometimes absent. The major FAs in all species were DHA and EPA (Table 13). The contents of arachidonic and other n6 acids were generally low, 0–1.4%, and the n6/n3 ratio was no greater than 0.1%. Only *Aequorea victoria* was distinguished by marked quantities of both 20:4n6 and 22:5n6. In most species, the presence of high levels of such acids as 20:1, 22:1, and 18:1n9 was associated with carnivorous feeding.

## 6. Scyphozoa

The class Scyphozoa, or ‘true medusae’, includes the orders Coronatae (mainly deep-sea species), Rhizostomeae, and Semaeostomeae. The total number of species is greater than 220 [1]. The most commonly known scyphozoans of the genera *Aurelia*, *Cyanea*, and *Chrysaora* are usually found near beaches. Jellyfish range in size from a dozen of millimeters to more than two meters in diameter. Jellyfish often get caught in fishing gear and nets along with fish and then are discarded. Nevertheless, the jellyfish catch accounts for about 3% of total fish landing [68]. Jellyfish can be used as a sustainable source of high-value compounds with biotechnological applications [7]. The largest (in size) species is *Cyanea arctica*, whose tentacles may reach over 40 m in length [1]. All medusae are carnivorous, but one of the *Cassiopea* species, Rhizostomeae, is known to possess symbiotic zooxanthellae. The major PUFAs are best known and studied: EPA, DHA and AA. The proportion of C20:1+C22:1 was high, up to 17.8% (Table 14).

For *Atolla* species, Coronatae, the major FAs were EPA, DHA, and DPA3 (Table 15). They showed marked concentrations of the monoenoic acids 16:1n7, 18:1n7 and n9, and 20:1n9. These fatty acids are typical of carnivorous species. Three Rhizostomeae species had 18:0,18:1n9, EPA and DHA as major FAs and much lower contents of 16:1n7, 18:1n7 and C20:1 and C22:1 than those recorded from *Atolla*. The FA composition of the zooxanthellate *Cassiopea* looked somewhat different [73]. The presence of 18:3n6 and 18:4n3 indicated symbiotic dinoflagellates, but the PUFA content was low, only 18.4%. Also, there were very low concentrations of EPA and DHA.

Tetracosapolyenoic acids have long been known as characteristic of Octocorallia. They have been found as major acids in Alcyonacea, Gorgonacea, Pennatulacea, and Stolonifera. However, Helioporacea shows some distinguishing features. Specimens of this taxon collected from Vietnam had calcified skeletons of crystalline aragonite and contained only small amounts of 24:6n3 (almost 2.0%), which were the lowest for Cnidaria. I found four papers where TPAs were detected: *Catostylus tagi* contained only one TPA, 24:5n6 and *Pelagia noctiluca* had the rare acid 24:4n6, while *Aurelia aurita* and *Rhopilema asamushi* had mainly 24:6n3 (Table 16).

Recent data on the presence of tetracosapolyenoic acids in Scyphozoa medusae suggest a wider distribution of tetracosapolyenoic acids. As experiments have shown, TPA causes a more pronounced inhibition of the synthesis of triglycerides and sterol esters than EPA and DHA [78]. The hexacosapolyenoic acids (HPAs) 26:5n3, 26:6n3, and 26:7n3 were found in *Rhopilema asamushi*. Their chemical structure was confirmed by GC–MS, and their total content in *Rh. asamushi* was 1.6% [75]. All members of this group had high proportions of PUFAs (48.3–66.0%); the n3/n6 ratio was 2.6–21.1. The total proportion of tetracosapolyenoic acids in Scyphomedusae was 3.1–10.4%.

## 7. Summary

This review provides an overview of the available data on fatty acid (FA) compositions for various cnidarian taxa. Obviously, for reliable results, standard methods are necessary that allow us to analyze a very extensive set of C12–C28 fatty acids and those with 1–7 double bonds. Otherwise, some important FAs can be omitted because of too long retention times, like C24–C26 PUFAs or demospongic acids C24–C28 with 2–3 double bonds, or can be destroyed, like 18:5n3 in basic transesterification.

### 7.1. Class Hexacorallia

In Actiniaria species, the major PUFAs were DHA, 22:5n3 (DPA3), EPA, and 22:4n6, with n3 FAs prevailing over n6 FAs. The content of arachidonic acid was relatively low, 2.6–7.7%, and was always lower than EPA. The total amount of C22 PUFA was higher than that of C20 PUFA. Abyssal and cold-water species accumulated monoenoic C20:1 and C22:1 acids. Together with noticeable concentrations of 18:1n9 and DHA, they indicated feeding on zooplankton. Of particular interest are the markedly high values of 22:5n3 and 22:4n6.

The order Antipatharia: *Stauropathes arctica* showed a somewhat simplified FA composition, the highest levels of C20 and C22 monoenoic acids (34.6%), EPA and DPA3 as major PUFAs (with a total of 27.7%), and surprisingly low contents of AA and DHA. The zoantharian genus *Palythoa* showed the dominance of the n6 acids AA and 22:4n6 and only traces of C20:1 and C22:1. The presence of 18:3n6, 18:4n3, and even 18:5n3 indicated the contribution of symbiotic zooxanthellae. The input of bacterial FAs was also noticeable.

The order Scleractinia: To date, these corals have been quite comprehensively investigated. The major unsaturated FAs in them were 18:1(n-9), 20:4n6, 20:5n3, 22:4n6, and 22:6n3. Some of the coral families had significant levels of characteristic FAs: 20:3n6 for Pocilloporidae, 18:3n6, 18:4n3, and 22:4n6 for Acroporidae, and 18:3n6 for Poritidae. The noticeable amounts of 18:3n6 and 18:4n3 indicated the input of symbiotic microalgae. The asymbiotic specimens of *Tubastrea* showed a significant difference from the symbiotic specimens. The contents of major FAs such as 18:1n9 (23.3–26.4%), 20:5n3 (10.9–14.9%), and 22:5n6 (16.4–17.3%) were multifold higher than in the symbiotic species. Also, the content of 22:6n3 was surprisingly low (only 1.3–1.4%), while in the symbiotic species, the level of DHAs ranged between 5.3 and 16.9%.

### 7.2. Class Octocorallia

The fundamental difference between Octocorallia and Hexacorallia consists of the presence of the tetracosapolyenoic acids 24:6(n3) and 24:5n6 in the former. These FAs are common and major components of Alcyonacea, Gorgonacea, Helioporacea, Pennatulacea, and Stolonifera.

The order Alcyonacea: This order was characterized by the dominance of n6 acids (with the n6/n3 ratio being 2–3). In all species, the major PUFAs were AA, 18:3n6, DHA, EPA, 24:5n6, and 18:4n3. The high concentrations of the FAs 18:3n6 and 18:4n3 indicated the major role of zooxanthellae in the food balance in Alcyonacea. The levels of bacterial odd- and branched chain acids in Lobophytum, Sarcophytum, and Dendronephthya were unusually high. The asymbiotic species had the highest concentration of tetracosapolyenoic acids, 10.0%. The absence of zooxanthellae and lack of TPA in any available food suggest the biosynthesis of TPA in Octocorallia.

The order Gorgonacea: Most Gorgonacea do not have zooxanthellae. They were characterized by marked contents of major PUFAs such as arachidonic acid (up to 47.6% in *Echinogorgia* sp.) and TPA (with a total of 4.5–21.1%, mainly 24:5n6). The low levels of EPA, C20:1 and 22:1 and a noticeable concentration of bacterial FA suggest the suspension feeding mode. Zooxanthellate *Rumphella* species were distinguished by the presence of FAs typical of symbiont microalgae, 18:3n6 and 18:4n3, and a lower proportion of TPA. The FA composition of *Bebryce* sp. with an unusual sponge symbiont included 17.2% of the demospongic acids Δ5,9-25:2, Δ5,9-26:2, Δ5,9,19-26:3, and Δ5,9,19-28:3, which are major FAs in marine and freshwater sponges of the class Demospongiae [53]. Moreover, this species had 8.8% of odd- and branched-chain bacterial FAs.

The order Helioporacea is unique among octocorals in producing calcified skeletons of crystalline aragonite. Lipids of *Heliopora* had high concentrations of the saturated acids 16:0 and 18:0 and an unexpectedly low content of AA, less than 1%. Another feature of the FAs from *H. coerulea* was the lowest content of tetracosapolyenoic acids, with approximately 2% of 24:6n3. The major PUFAs in *Carijoa riisei* (azooxanthellate) and *Clavularia* sp. were AA, 18:4n-3, EPA, DHA, and 24:5n-6. The noticeable proportion of 18:3n6 and 18:4n3 suggests the essential role of zooxanthellae.

The order Pennatulacea: There are plenty of data on the FA composition of Pennatulacea species, but most of the publications do not report the presence of the tetracosapolyenoic acids 24:5n6 and 24:6n3, which are major components (with a total amount of up to 20%). In the species with detected TPA, the rest of the PUFAs were EPA, AA, 22:4n6, a low level of DHA, and noticeable levels of C20:1 and C22:1. A comparative analysis of 25 sea pen samples showed that these animals apparently consumed more diatoms and/or zooplankton.

### 7.3. Class Medusozoa

The order Cubomedusae is a non-speciose axon of the phylum Cnidaria, also known as box jellyfish, usually small in size. The data on FAs in cubozoans are limited to a relatively old communication. The major PUFAs were the n3 family: 22:5n3, EPA, and DHA. They had noticeable levels of the symbiotic acids 18:3n6 and 18:4n3, a high n3/n6 ratio (5.4–8.8), and low contents of C20:1 and C22:1 acids, which may be explained by the contribution of symbiotic algae and feeding on diatoms. *Maeotias inexpectata* differed by low levels of AA and EPA and an unexpectedly high content of AA and EPA homologs, the acids 22:4n6 (13.4%) and 22:5(n3) (21.2%).

### 7.4. Class Hydrozoa

The hydrozoan family Milleporiidae is commonly known as ‘stinging coral’ or ‘fire coral’. Millepores are voracious zooplankton feeders that can also obtain part of their nutrition from autotrophic sources, i.e., photosynthetic production by symbiotic zooxanthellae. The dominance of C22 PUFAs, especially DHA, 22:5n6 and 22:4n6, is characteristic of the *Millepora* species. In some species, C20:1 acid is present in noticeable amounts. In addition, they contain 18:3n6 and 18:4n3, characteristic of symbiotic microalgae. These data and the exceptionally high level of DHA suggest polytrophic feeding on zooplankton, microalgae, and symbiotic zooxanthellae.

*Physalia* (from the family Siphonophora) and *Velella* (Anthoathecata) are pleustonic hydrozoan genera. In both, DHA, EPA, and 18:4n3 as major PUFAs and a very low content of n6 acids were found. The n3/n6 ratios were high, 10.8 and 12.4, respectively. The presence of C18 PUFA suggests the existence of the zooxanthellae symbiont. However, two previous studies on *Physalis* showed AA as a major acid, and the n3/n6 ratios were 1.0 and 1.7. The low values of C20:1 and C22:1 and the high concentrations of DHA indicated feeding on microalgae.

In the Hydromedusae families Thecata, Rhopalonematidae, Bythotiaridae, and Physaliidae, the major FAs were DHA and EPA. The contents of arachidonic and other n6 acids were generally low, 0–1.4%, and the n6/n3 ratio was no greater than 0.1%. Only *Aequorea victoria* differed by marked contents of both 20:4n6 and 22:5n6. In most species, the presence of high levels of such acids as 20:1, 22:1 and 18:1n9 could be associated with carnivorous feeding.

### 7.5. Class Scyphozoa

The class Scyphozoa, or ‘true medusae’, consists of the orders Coronatae (mainly deep-sea species), Rhizostomeae, and Semaeostomeae. All medusae are carnivorous, but one of the *Cassiopea* species (Rhizostomeae) is known to possess symbiotic zooxanthellae. The FA compositions of common medusae from the order Semaeostomeae were characterized by such major components as EPA, DHA and AA. Low contents of C18 PUFA and significant levels of C20:1 andC22:1 and 18:1n9 are typical of carnivorous feeding. *Atolla wyvillei* (Coronatae) showed an FA composition similar to that of the Semaeostomeae species. The rest of the Rhizostomeae species demonstrated the lack of C20:1 and C22:1 acids, which may be explained by feeding on microalgae and unspecific suspended particulate matter in the water column.

Tetracosapolyenoic acids have long been known as characteristic of Octocorallia. Nevertheless, I found four papers where TPAs were reported for medusae: *Catostylus tagi* contained only one TPA, 24:5n6 and *Pelagia noctiluca* had the rare acid 24:4n6, while *Aurelia aurita* and *Rhopilema asamushi* had mainly 24:6n3. These data on the presence of tetracosapolyenoic acids in Scyphozoa medusae suggest a wider distribution of tetracosapolyenoic acids. The total proportion of tetracosapolyenoic acids in Scyphomedusae ranged between 3.1 and 10.4%.

In this review, I presented contemporary data on fatty acids in the main taxa of the ancient marine phylum Cnidaria. Numerous cnidarian species inhabit very different biotopes, from tropical and arctic seas to abyssal depth. This investigation on FA distribution in cnidarians gives information on the influence of many factors such as the taxonomy, depth, temperature, feed and presence of symbionts on the lipid and FA biochemistry of Cnidaria.

## Figures and Tables

**Table 1:** Main FA composition of total lipids of Actiniaria species (% of total FA).

Fatty Acids	*Bathyphelli* *australais*	*Metridium* *dianthus*	*Metridium* *senile*	*Aiptasia* *pallida*	*Actinia* *equina*	*Anemonia* *viridis*	*Actinostola* *callosa*
16:0	9.7	4.6	12.6	21.2	28.5	13.7	7.9
16:1n7	1.5	0.6	3.3	3.0	3.5	0.5	2.5
18:0	3.2	5.9	9.9	7.7	11.3	9.1	6.1
18:1n9	9.3	2.7	9.2	5.3	8.5	1.4	5.9
18:1n7	5.5			1.1	7.1	1.1	
18:2n6	0.4		1.1	3.2	2.1	0.3	0.6
18:3n6			2.8	7.8		0.8	
18:4n3	1.1					1.4	
20:1n7	7.7	7.3				3.6	2.3
20:1n9	2.0				2.2	6.4	10.8
AA	3.1	2.6	7.7	4.80	2.8	5.8	
EPA	14.0	27.1	12.5	4.1	9.1	28.7	14.1
22:1	3.4	4.9	0.8				14.6
22:4n6	2.0	4.2	8.6	8.0	0.3	8.1	5.9
22:5n3	6.0	12.0	1.2	3.6		12.3	9.3
DHA	11.5	19.0	17.9	22.9	5.2	0.3	9.9
18:1n9/n7	1.7	NA	NA	5.0		1.3	NA
n6/n3	0.11			0.79	0.58	0.4	0.27
20:1+22:1	13.5	4.9	0.8		2.4	10.0	27.5
References	[25] abyssal	[26] abyssal	[27] littoral	[28] tropical	[29] temperate	[30] aquarium	[31] batial

**Table 2:** Major fatty acids of Antipatharia and Zoantharia corals (as % of total FAMEs).

Fatty Acids	Antipatharia *Stauropathes arctica*	Zoantharia *Palythoa* 5 Species	Zoantharia *Palythoa caesia*	Zoantharia *P. caribaeorum*
16:0	6.42	18.1–26.4	29.5	43
16:1n7	1.85	3.4–9.9	4.3	3.3
18:0	1.35	14.9–4.5	9.9	6.2
18:1n9	6.01	4.0–8.9	3.9	1.4
18:1n7	6.95	6.5–1.5	1.4	1.2
18:2n6	0.47	0.8–2.9	1.1	
18:3n6	0.04	1.7–3.6	2.3	
18:4n3	0.79	0.9–3.8	3.2	4.9
18:5n3		+	0.42	
20:1n9	14.47	0.5–1.5	0.7	0.8
20:1n7	1.85		0.2	
20:4n6	1.48	1.2–15.0	10.3	5.2
20:5n3	10.61	–	3.4	
22:6n3	1.61	0.0–1.9	2.3	
22:5n3	17.08	3.8–10.1	6.5	3.0
22:4n6	1.39	1.2–15.0	4.6	4.8
22:1n9	7.48		0.3	0.5
22:1n11(13)	8.65			
C20:1+C22:1	34.6		1.6	
Odd + Br	1.1	5.3–8.9		5.8
16:1n7+18:1 n7	8.8	5.1–17.4	5.7	5.5
References	[39]	[40]	[41]	[34]

**Table 4:** Total FA composition (as % of total lipids FAME) in scleractinian corals from the families Agariciidae (mean of 2 species), Dendrophylliidae (mean of 5 species), Euphylliidae (mean of 2 species), Faviidae (mean of 5 species), Fungiidae (mean of 3 species), and Pectiniidae (mean of 2 species) [9,48].

	Agariciidae	Dendrophylliidae		Euphylliidae	Faviidae	Fungiidae	Pectiniidae
Fatty Acids	*Pavonaria*	*Turbinaria ^a^*	*Balanophillia* sp. *^a^*	*Euphyllia*	*Favia*	*Fungia*	*Echinophyllia*
16:0	37.3	32.6	9.5	38.3	40.4	46.7	35.1
16:17	2.2	3.0	3.2	3.7	3.7	1.8	3.9
18:0	7.8	6.4	6.1	5.8	4.5	8.6	5.6
18:19	6.0	4.6	20.5	6.0	7.6	4.2	8.3
18:1n7	1.0	1.1	3.4	1.0	1.1	0.7	1.2
18:2n6	1.4	2.1	1.5	2.2	1.6	1.5	3.1
18:3n6	6.5	11.2	0.9	6.3	10.8	6.4	8.3
18:4n3	1.6	3.1	0.6	1.6	1.2	1.1	1.9
20:1n9	2.4	0.2	1.9	0.3	0.4	0.4	0.7
20:3n6	2.7	1.7	1.1	3.9	1.9	2.2	2.7
20:4n6	6.7	8.4	10.9	11.2	4.6	5.5	6.4
20:5n3	1.9	2.0	7.3	2.5	0.8	1.8	1.5
22:4n6	2.9	3.3	6.7	2.6	2.1	2.9	1.9
22:5n3	2.3	4.6	13.0	0.4	6.7	2.6	5.5
22:6n3	7.9	7.2	1.7	8.6	3.6	6.9	5.5
n6/n3	1.6	1.6	1.0	1.0	1.7	1.5	1.5

*^a^* Azooxanthellate species.

**Table 5:** Main FA composition of total lipids in Alcyonacea (% of total FA) [50] (sp—means number of investigated species).

	Alcyoniidae					Nephtheidae	
FA	*Klyxum molle*	*Cladiella* 3 sp.	*Lobophytum* 5 sp.	*Sarcophytum* 6 sp.	*Sinularia* 12 sp.	*Nephthea*	*Dendroneph thya* 3 sp. ^a^
16:0	13.60	34.60	29.24	29.29	27.43	20.30	28.67
16:1n7	5.20	4.00	2.64	2.87	3.15	0.90	2.17
16:2n7	0.40	0.30	5.92	9.43	4.74		4.43
18:0	6.00	8.30	6.06	5.29	6.22	15.80	8.60
18:1n9	5.00	4.90	2.46	3.16	2.38	4.10	3.10
18:1n7	0.30	0.40	0.70	0.57	0.43	0.10	0.87
18:2n6	1.70	2.60	0.45	0.20	1.02	2.40	1.00
18:3n6	10.60	9.40	0.17	0.37	7.40	7.90	0.40
18:3n3	0.20	0.10	0.24	0.77	0.31	0.20	0.33
18:4n3	8.70	3.70	2.86	4.30	2.79	2.60	1.97
20:1n9	0.20	0.20	0.16	1.05	0.15	0.20	0.30
20:4n6	19.70	9.40	19.50	18.27	15.78	18.00	15.33
20:4n3	0.30	0.10	0.58	0.47	0.90	0.70	0.87
20:5n3	4.80	2.10	1.96	1.53	2.78	5.90	1.43
22:5n6	0.10	0.10	0.30	0.27	0.23	0.20	1.10
22:6n3	6.90	5.20	2.10	2.50	4.82	3.20	2.47
24:5n6	5.50	2.20	4.82	5.63	4.98	4.80	8.40
24:6n3	1.20	0.90	1.18	0.83	1.94	1.60	1.63
C24 PUFA	6.70	3.10	6.00	6.46	6.93	6.40	10.03
n6/n3	1.73	1.99	2.88	2.39	2.25	2.49	2.94
Odd+Br	1.10	0.60	8.28	3.90	2.52	0.57	5.60

^a^—azoxanthellate species.

**Table 7:** Main FA (% of total FAMEs) composition of azooxanthellate, zooxanthellate species of Gorgonacea and *Bebryce* sp. with a sponge symbiont [51].

Fatty Acids	Azooxanthellate Gorgonacea Mean of 6 Species	Zooxanthellate *Rumphella aggregata*	*Bebryce* sp. with Sponge Symbiont
16:1n7	1.54	2.65	1.9
16:0	10.36	32.95	8.9
18:3n6	0.1	1.1	-
18:4n3	0.21	2.15	0.3
18:2n6	0.96	0.65	0.7
18:1n9	2.93	3.85	1.8
18:1n7	2.05	0.35	2.1
18:0	6.08	9.6	5.4
20:4n6	40.5	13.15	21.7
20:5n3	2.45	2.05	2.0
22:5n6	1.65	0.1	4.2
22:6n3	2.35	1.6	3.6
22:4n6	3.07	0.65	1.1
24:5n6	9.24	3.4	7.2
24:6n3	2.16	0.2	0.5
n6/n3	7.5	2.9	5.3
Odd & Br	4.55	3.0	6.1
20:1+22:1	1.07	0.6	1.2
Demospongic acids *			17.2%

* Demospongic acids: Δ5,9-25:2, Δ5,9-26:2, Δ5,9,19-26:3, and Δ5,9,19-28:3.

**Table 8:** Major fatty acids (% of total FAMEs) in Helioporacea (*Heliopora coerulea*) and Stolonifera (*Clavularia* sp. and *Carijoa riisei*).

Fatty Acids	*H. coerulea*	*H. coerulea*	*Clavularia* sp.	*Carijoa riisei*	*Carijoa riisei*
16:0	40.9	45.5	14.7	7.4	20.4
16:1n7	3.1	2.3	1.6	0.7	0.6
18:0	7.3	6.6	2.7	4.7	24.6
18:1n9	3.1	2.4	4.0	4.9	8.2
18:1n7	0.2	0.8	0.3	-	0.2
18:2n6	2.1	3.2	6.3	1.8	1.6
18:3n6	15.1	1.0	0.5	9.4	4.8
18:4n3	3.5	2.7	8.3	10.6	4.6
20:3n6	0.9	0.3		0.9	0.9
20:4n6	0.2	0.6	21.6	17.7	7.4
20:5n3	5.4	10.2	7.5	11.4	5.8
22:4n6	0.4	-		0.7	0.2
22:5n3	0.5	1.8	2.6	-	0.1
22:6n3	4.7	9.9	10.5	7.3	3.2
24:5n6	-	-	2.4	8.9	4.0
24:6n3	1.7	2	0.4	2.1	0.8
References	[54]	[48]	[48]	[48]	[48]

**Table 9:** Pennatulacea FA composition (% of total FAMEs) of genera *Pennatula*, *Pavonaria*, *Malacobelemnon*, and *Veretillum*.

FA	*Pennatula aculeate* (3 Specimens)	*Pavonaria finmarchica* PL	*P. finmarchica* NL	*Rennilla koellikeri* (2 Specimens)	*Veretillum cynomorium* (6 Specimens)	Sea Pens (25 Species)
16:0	7.3–8.9	4.9	8.7	16.1–17.7	8.3–10.9	12.6
16:1n-7	3.2–4.7	0.9	5.0	1.6–2.3	1.2–1.8	4.3
17:0	0.6–0.7	0.4	2.2	2.2–3.0	1.2–1.4	
18:0	2.1–2.6	1.3	0.6	8.0–9.5	5.9–6.8	2.6
18:1n-9	5.6–9.7	2.3	11.3	1.8–2.2	1.2–2.7	10.2
18:1n-7	5.1–7.2	–	–	3.3–4.2	2.0–2.7	3.7
20:1n-9	12.8–14.0	12.2	10.3	0.9–1.8	0.4–0.6	8.1
20:1n-7	3.0–3.4				0.7–1.1	
20:4n6	9.0–15.7	4.3	1.2	31.4–41.9	6.2–10.0	4.6
20:5n3	8.5–15.7	38.3	19.8	4.4–18.3	9.0–12.4	17.1
22:1n-11	10.9–11.4	0.3	2.1			8.2
22:1n-9	4.3–4.7				0.2	4.8
22:4n6	5.5–8.9	0.6	0.3		2.5–3.9	1.2
22:5n3	2.2–2.7	0.3	0.4		0.9–1.3	1.2
22:6n3	1.0–2.2	2.5	9.4	0.7–1.3	0.8–2.2	3.4
24:5n6	ND	6.0	3.6	ND	0.8–1.0	ND
24:6n3	ND	18.0	16.4	ND	7.7–11.5	ND
n3/n6		4.8	6.7	0.21–0.48	2.02–2.88	3.04
C20:1+C22:1	18.2–19.5	12.5	12.4			24.48
References	[31] deep sea	[36] deep sea	[36] deep sea	[58] shallow water	[59] shallow water	[39] deep sea

**Table 10:** Major FA (% of total FAMEs) in the cubozoan families Olindiidae, Bougainvilliidae, Chirodropidae, and Tamoyidae [60].

FA	*Maeotias inexpectata*	*Nemopsis bachei*	*Chiropsalmus* sp.	*Tamoya haplonema*
16:0	9.5	8.5	17.6	8.5
16:1	2.6	1.8	4.9	2.0
18:0	10.1	9.1	10.4	9.3
18:1	4.7	3.7	6.6	6.6
18:2n6	1.0	1.2	1.2	1.2
18:3n3	0.4	2.0	0.8	0.5
18:4n3	0.5	2.3	0.23	0.7
20:0	0.3	1.7	0.3	0.2
20:1	0.8	2.0	1.3	1.1
22:1	0.5			0.5
AA	1.3	3.3	3.3	5.0
EPA	2.6	19.1	17.6	18.4
22:4n6	13.4	2.5	3.1	3.3
22:5n6	1.6	0.7		0.7
22:5n3	21.2	14.7	17.6	18.4
DHA	16.3	20.0	6.7	15.3
(n3)/n6	2.4	8.8	7.6	5.4

**Table 11:** Major FA composition (% of total FAs) of hydrozoan corals of the genus *Millepora* from Vietnam (V) and the Seychelles (S) [47,62].

Fatty Acid	*Millepora* sp. V	*M. platyphylla* V	*M. dichotoma* V	*M. platyphylla* S	*M. dichotoma* S	*M. murrayi* Okinawa
16:0	6.3	23.6	19.8	17.6	18.9	29.0
16:1n7	-	0.1	-	0.2	-	1.0
18:0	7.1	15.4	15.3	21.3	19.4	17.4
18:1n9	1.4	6.1	3.9	2.4	3.2	2.8
18:2n6	0.6		0.1`	0.4	0.7	2.1
18:3n6		0.2		0.2	0.4	2.4
18:4n3	6.9	1.5	1.9	5.2	4.8	10.8
20:0		3.3	5.5	-	-	-
20:1n9	0.3	0.2	0.4	6.3	6.1	-
20:4n6	0.4	0.7	-	0.3	1.0	0.3
20:5n3	1.1	0.4	0.8	0.4	0.6	-
22:4n6	3.8	2.6	3.5	4.6	3.7	-
22:5n6	8.3	6.8	7.3	10.0	8.5	-
22:5n3	-	0.4	1.1	0.7	0.9	0.3
22:6n3	61.5	32.0	33.3	27.5	28.0	1.7
Saturated	14.0			39.0	40.0	62,2
PUFA	83.9			51.0	49.6	21.4
n3/n6	5.1	2.3	3.3	2.0	2.3	

**Table 12:** FA composition (% of total FAs) of the hydrozoan families Physaliidae and Velellidae.

Fatty Acids	*Physalia physalis*	*Physalia physalis*	*Physalia physalis*	*Velella velella*
16:0	22.6	17.5	22.4	16.0
16:1n7	3.9	1.2	0.8	1.2
7M7-16:1	3.2	6.1		
17:0	1.0	1.1	1.2	0.4
18:0	9.1	15.4	9.9	5.0
18:1n9	5.1	3.4	4.9	7.3
18:1n7	1.6	0.9	1.2	0.5
18:2n6	1.4	0.6		1.2
18:3n6	0.5	0.8		0.9
18:3n3	0.9	0.4		1.0
18:4n3	1.2	0.2	2.1	3.8
20:1n9	0.6	1.2	0.5	4.2
AA	9.1	13.2	0	0.3
20:3n3		0.1	2.3	0.1
EPA	7.7	5.4	6.5	7.8
22:4n6	0.7	2.2	0.3	0.5
22:5n6	4.3	6.7	1.6	0.5
22:5n3	1.1	1.4	1.9	1.4
DHA	15.5	16.2	22.9	27.6
n3/n6	1.7	1.0	12.5	10.9
Reference	[64]	-	[63]	[63]

**Table 13:** Main FA composition (% of total FAs) of the Hydromedusae families ^a^ Thecata, ^b^ Rhopalonematidae, ^c^ Bythotiaridae, and ^d^ Physaliidae.

FA	*Aequorea victoria* ^a^	*Arctapodema ampla* ^b^	*Calycopsis borchgrevinki* ^c^		*Dimophyes arctica* ^d^	*Diphyes antarctica* ^d^	
16:0	7.7	14.9	16.6	11.6	18.9	18	20.1
18:0	8.9	5.1	4.8	7.8	9.3	8.8	7.6
16:1n7	2.6	2.8	9.2	5.2	15.3	4.0	3.8
18:1n9	5.4	5.7	26.4	14.3	17.2	8.7	7.3
18:1n7	1.1	2	3.7	1.9	4.1	2.0	4.1
20:1n9	2.1	1.8	2.5	4.4	1.1	1.2	1.7
20:1n7	3.1	2	2.8	6.9	1.0	3.6	0.6
22:1n9	6.2	2.7	0	0.3	1.3	0.1	0
18:2n6	0.4	0.9	1.5	0.9	1.0	2.2	1.9
18:4n3	0.4	0.2	0.1	0.1	0.2	2.1	0.6
20:4n6	9.6		1.4		0	1.4	1.2
20:5n3	9.6	16.4	9.6	9.9	8.2	16.5	19.1
22:4n6	0.3			2.1			
22:5n6	4.4						
22:5n3	1.5	0.2	2.1	5.6	0.1	0.2	0.3
22:6n3	18.8	26.4	7.0	16.1	10.4	16.9	17.6
Odd+Br	1.7	1.7	0.3	1.2	0.8	1.1	1.0
20:1+22:1	11.4	6.5	5.3	12.5	3.4	5.1	2.3
n6/n3	0.17	0.02	0.08	0.09	0.05	0.06	0.05
References	[65]	[66]	[67]	[66]	[67]	[67]	[67]

**Table 14:** Major FA compositions (% of total FAs) of the Scyphozoa order Semaeostomeae.

FA	*Aurelia aurita*	*Aurelia aurita*	*Cyanea lamarckii*	*Cyanea capillata*	*Chrysaora isosceles*
14:0	2.3–3.3	3.3	3.5	1.4–2.2	3.3
16:0	21.7–28.8	16.0	19.0	10.1–14.4	9.5
17:0	1.3–2.8		1.0	1.8–2.1	0.6
18:0	12.5–17.8	6.4	12.0	4.5–7.1	7.1
16:1n7	3.1–6.0	4.6	4.7	2.3–5.2	3.7
18:1n9	1.4–2.6	8.9	5.5	4.6–7.2	4.4
18:1n7	2.2–2.4	2.6	3.1		1.5
20:1	0.3–0.6	20.4.8	3.4	6.8–12.5	6.6
22:1	3.0–10.0	3.2	2.5	1.9–2.4	6.1
18:3n3	0.6–0.8	4.5	0.4	0.3–0.8	0.6
18:4n3	0.4	0.1	0.6	0.5–0.6	1.3
20:4n6	2.8–8.6	6.7	8.7	6.2–9.1	5.4
20:5n3	10.2–15.6	8.5	13.8	9.8–19.4	20.0
22:4n6		0.6		0.9–2.0	
22:5n6				0.2–1.8	
22:5n3	1.3–2.5	0.3	3.2	2.0–4.8	5.4
22:6n3	3.0–6.1	7.0	12.1	10.4–20.4	19.7
C20:1+C22:1	3.3–10.6	17.8	5.9	9.2–14.4	12.7
Reference	[69]	[70]	[71]	[72]	[71]

**Table 15:** Major FAs (% of total FAs) of the Scyphozoa orders Coronatae and Rhizostomeae.

FA	Coronatae	Rhizostomeae			
	*Atolla wyvillei*	*Cotylorhiza tuberculata*	*Rhizostoma octopus*	*Phyllorhiza punctata*	*Cassiopea* sp. ^Z^
14:0	2.0–6.1	2.9	5.1	7.7	3.1
16:0	16.0–20.8	26.1	27.3	26.0	26.7
18:0	2.8–11.9	24.2	21.7	17.7	5.4
16:1n7	5.3–2.6	1.2	3.8	3.8	1.9
18:1n9	11.8–9.0	12.8	6.8	9.3	7.25
18:1n7	7.2–4.1	1.2	3.5		
20:1n9	4.6–7.3		0.8		
22:1n9	0.4–0.5		-		1.5
18:2n6	2.5–1.4	8.3	1.6	2.4	1.65
18:3n3	1.0–0.5		1.6	1.2	
18:3n6					0.9
18:4n3	0.9–5.0		2.8		4.1
20:3n6				5.1	
20:4n6	0–4.2	5.3	2.8	6.2	4.5
20:5n3	16.6–15.0	5.1	9.7	6.3	1.0
22:5n3	2.4–9.7		1.3		1.0
22:6n3	5.6–3.9	7.2	5.3	7.4	2.6
C20:1+C22:1	4.8–7.8				
Reference	[67]	[72]	[70]	[7]	[73]

^Z^ Zooxanthellate species.

**Table 16:** Major FA composition (% of total FAs) of Scyphozoa medusae with C24 and C26 PUFAs.

Fatty Acids	*Aurelia aurita*	*Aurelia aurita*	*Rhopilema asamushi*	*Catostylus tagi*	*Pelagia noctiluca*
20:4n6	9.9 ± 2.3	1.84 ± 0.06	8.39 ± 3.85	7.5	18.5–9.0
20:5n3	14.1 ± 1.9	33.28 ± 0.80	13.05 ± 3.90	13.5	14.6–10.1
22:6n3	9.8 ± 1.6	11.20 ± 1.25	12.29 ± 1.97	11.2	9.7–5.6
22:4n6	0.6 ± 0.0	0.26 ± 0.02	2.35 ± 1.96		4.0–2.4
22:5n3	1.1 ± 0.2	5.03 ± 0.41	5.07 ± 1.54	2.1	
24:5n6	1.1 ± 0.2	0.00 ± 0.00	0.44 ± 0.24	3.9	
24:6n3	9.3 ± 1.8	9.48 ± 1.00	6.00 ± 1.96		
24:4n6			0.69 ± 0.62		2.4–1.1
24:5n3		0.10 ± 0.01	0.31 ± 0.06		5.3–2.0
24:4n3			0.47 ± 0.17		
26:7n3			0.55 ± 0.27		
26:6n3			0.44 ± 0.16		
26:5n3			0.63 ± 0.39		
(n3)/n6	2.61	21.06	3.09	3.7	
∑C24 PUFA	10.4	9.58	7.90	3.9	7.7–3.1
∑C26 PUFA			1.62		
References	[74]	[75]	[75]	[76]	[77]

## Data Availability

The data presented in this study are available on request from the corresponding author.
